# Elevated Th22 Cells Correlated with Th17 Cells in Peripheral Blood of Patients with Acute Myeloid Leukemia

**DOI:** 10.3390/ijms15021927

**Published:** 2014-01-27

**Authors:** Shuang Yu, Chuanfang Liu, Lei Zhang, Baozhong Shan, Tian Tian, Yu Hu, Linlin Shao, Yuanxin Sun, Chunyan Ji, Daoxin Ma

**Affiliations:** 1 Department of Hematology, Qilu Hospital, Shandong University, Jinan 250012, China; E-Mails: yussdu@gmail.com (S.Y.); liucfsy@gmail.com (C.L.); tiantian0621010155@gmail.com (T.T.); shaollsy@gmail.com (L.S.); sunyxsdu@gmail.com (Y.S.); jichunyan@sdu.edu.cn (C.J.); 2 Department of Orthopedics, Qianfoshan Hospital Affiliated to Shandong University, Jinan 250012, China; E-Mail: zhanglsdu@gmail.com; 3 Department of Stomatology, Jinan Central Hospital Affiliated to Shandong University, Jinan 250013, China; E-Mail: sbzsdu@gmail.com; 4 Department of Oncology, Qilu Hospital, Shandong University, Jinan 250012, China; E-Mail: huysdu@gmail.com

**Keywords:** acute myeloid leukemia, T helper 22, T helper 17, T helper 1, interleukin-22, interleukin-17

## Abstract

Acute myeloid leukemia (AML) is a hematological tumor in which progress T helper (Th) subsets including Th22, Th17, and Th1 cells play a pivotal role. However, the role of T helper (Th) subsets in the immune pathogenesis of AML remains unclear. Here, we investigated frequencies of Th22, Th17, pure Th17, and Th1 cells in the peripheral blood (PB) of AML patients. We demonstrated that Th22, Th17, and pure Th17 in newly-diagnosed (ND) and non-complete remission (Non-CR) AML patients and plasma IL-22 in ND AML patients were significantly increased. Retinoid-related orphan receptor C (*RORC*) expression was significantly elevated in CR and Non-CR AML patients. However, Th1 in ND AML patients and IL-17 in ND, Non-CR or CR AML patients was significantly decreased compared with controls. Moreover, Th22 and IL-22 showed positive correlation with pure Th17, but Th22 showed negative correlation with Th1 in ND AML patients. *RORC* showed positive correlation with Th22 and approximately positive correlation with pure Th17 in Non-CR patients. PB blast cell showed positive correlation with Th22 and negative correlation with Th1 in ND AML patients. Our results indicate that Th22 and pure Th17 cells conjointly contribute to the pathogenesis of AML and might be promising novel clinical index for AML.

## 1. Introduction

Acute myeloid leukemia (AML) is a disease with malignant clone of hematopoietic stem cells, characterized by the accumulation of considerable immature myeloblasts in bone marrow. Although abnormalities of immune system of AML have long been recognized [1–3], mechanism for the involvement of immune system impairment in the pathogenesis of AML remains unknown. Recent studies have shown that leukemic cells suppress the host immune system with tumor progression connected to immune cells dysfunction [4,5].

Th17 cells are novel CD4^+^ T cells that are characterized by the production of interleukin-17 (IL-17) [6]. The human *RORC* (retinoid-related orphan receptor C) has been considered as the essential transcription factor for Th17 differentiation [7]. IL-17, the main effector cytokine of Th17 cells, is responsible for inflammatory and autoimmune diseases [8,9]. Accumulating evidence indicates that IL-17 has tumor-promoting effects, especially in the context of inflammation [10–12]. Some studies in animals have also indicated that IL-17 may promote angiogenesis and tumor growth [13–15]. Currently, the association of Th17 cells and IL-17 with AML remains unclear as some studies have found elevated levels in newly-diagnosed (ND) AML patients while others have shown normal Th17 levels in ND AML patients [3,5,15–17].

More recently, a unique Th22 subset is clearly separated from Th17 and other known Th subsets with a distinct identity with respect to gene expression and function [18]. Th22 cells are identified inflammatory CD4^+^ T cells that produce IL-22 but do not express IL-17 or IFN-γ [19–22]. In contrast to other T cells such as Th1, Th2, and Th17 cells, Th22 cells showed a stable and distinct expressing profile [18]. Expression of CCL20 and IL-23R [23] was absent in Th22 clones, which is different from Th17 cells. Recent studies indicate that IL-6 and TNF-α, along with the help of plasmacytoid DCs, can promote the Th22 phenotype [19]. The clonal stability, the selective expression of transcription factors, PDGF receptor and CCR-10 [19], and the fact that native T cells differentiate toward Th22 phenotype in the presence of IL-6 and TNF-α [19], provide strong evidence that Th22 cells represent a terminally differentiated and independent T cell subtype. It has been shown that Th22 cells play an important and complicated role in some inflammatory and autoimmune diseases [18,24].

IL-22 was the effector cytokine of Th22 cells and recently discovered as an IL-9-inducible, T-cell-derived cytokine that belongs to the IL-10 gene family [25,26]. It is known that IL-22 exerts its function by binding to a heterodimeric receptor consisting of the IL-10 receptor (IL-10R) β chain and the IL-22R [18]. IL-22 induces signal transduction and activators of transcription (STAT) activation in several cell lines, such as mesangial cells, lung and intestinal epithelial cells, melanoma, and hepatoma cells [26,27]. Recent studies show that IL-22 has also been implicated in the etiology of inflammatory and autoimmune diseases [25,28–30], myelodysplastic syndrome (MDS) [31] and T-cell acute lymphoblastic leukemia (T-ALL) [32]. However, what the frequencies and role of these Th subsets are in AML have not been completely clarified.

In this study, we investigated Th22 (CD4^+^IFN-γ^−^IL-17^−^IL-22^+^), Th17 (CD4^+^IL-17^+^), pure Th17 (CD4^+^IFN-γ^−^IL-22^−^IL17^+^), and Th1 cells (CD4^+^IFN-γ^+^), plasma IL-22 or IL-17 levels and mRNA expression of *RORC* in peripheral blood (PB) of AML patients. Their correlations with disease activity were also evaluated in the present study.

## 2. Results and Discussion

### 2.1. Elevated Th22 Cells and Plasma IL-22 Level in AML Patients

**Figure 1.**
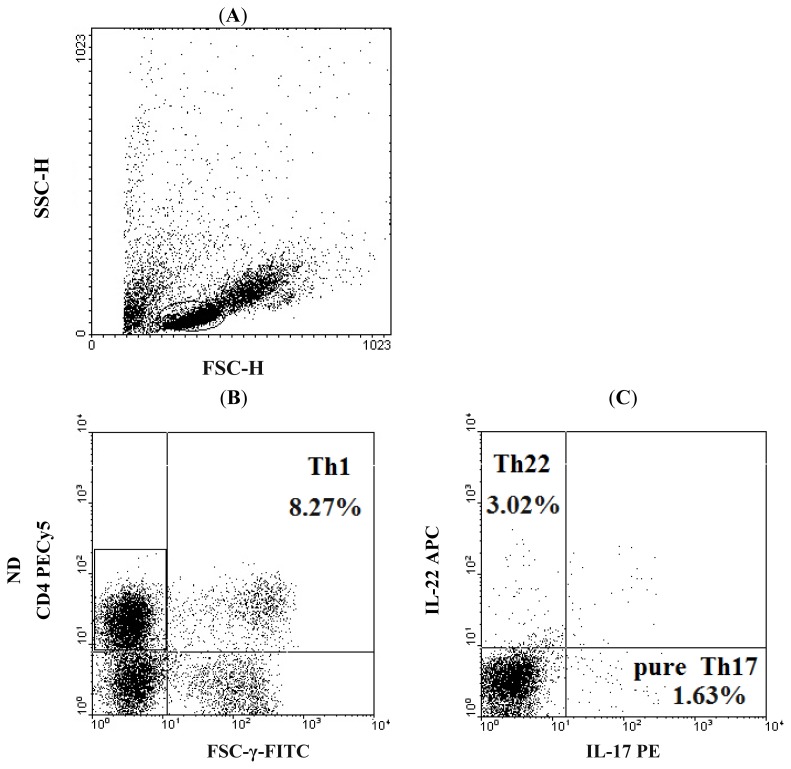

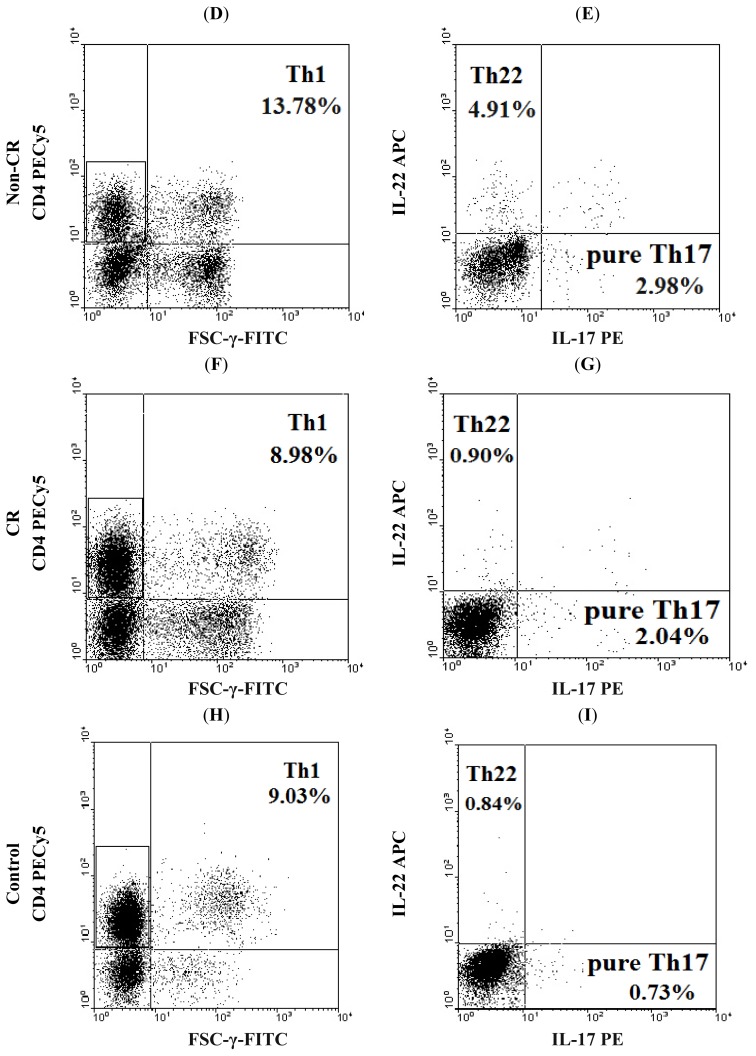
The percentages of circulating Th1, pure Th17, and Th22 cells in AML patients and healthy controls. (**A**) Lymphocytes were gated by flow cytometry; (**B**, **D**, **F**, and **H**) Plot in the rectangles represented CD4^+^ IFNγ^−^ T cells. The number in the quadrant represented circulating Th1 (CD4^+^IFN-γ^+^) cells from ND AML patients, Non-CR AML patients, CR AML patients and healthy controls; (**C**, **E**, **G**, and **I**) Representative IL-22 and IL-17 expression in CD4^+^ IFNγ^−^ T subsets from ND AML patients, Non-CR AML patients, CR AML patients and healthy controls. The percentages of circulating Th22 (CD4^+^IFN-γ^−^IL17^−^IL-22^+^) and pure Th17 (CD4^+^IFN-γ^−^IL-22^−^IL17^+^) cells were shown in the upper left and lower right quadrants, respectively.

**Figure 2.**
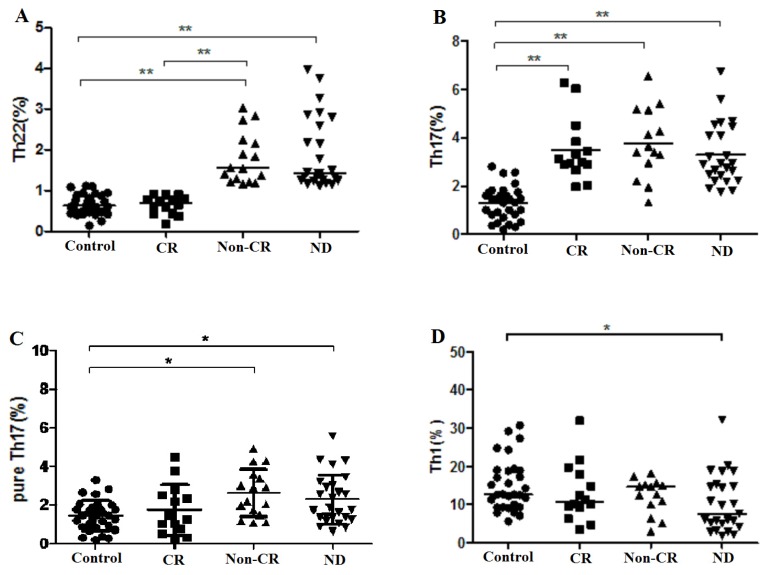
The results of circulating Th subsets in healthy controls, CR AML patients, Non-CR AML patients, and ND AML patients. (**A**) The percentages of circulating Th22 (CD4^+^IFN-γ^−^IL17^−^IL-22^+^) cells; (**B**) The percentages of circulating Th17 (CD4^+^IL-17^+^) cells; (**C**) The percentages of circulating pure Th17 (CD4^+^IFN-γ^−^IL-22^−^IL-17^+^) cells; (**D**) The percentages of circulating Th1 (CD4^+^IFN-γ^+^) cells. Data was shown as Median (range). * *p* < 0.05, ** *p* < 0.0001.

Recent research has delineated that defect of cellular immunity response may play a key role in the pathogenic mechanisms of AML. It is well known that persistent immunodeficiency is a common feature in patients with leukemia and T cell function becomes suppressed as the disease progresses [33,34]. Several Th cells, including Th1, Th17, and Treg have been largely investigated in AML. However, the hypothesis that these cells play key roles in progress of AML is insufficient to explain why so many immunological events happen early or after chemotherapy. Here, we first analyzed the percentage of Th22 cells from the cytokine patterns after *in vitro* activation by PMA/ionomycin in short-term culture. The expression of a typical dot plot of Th22 cells, defined as CD4^+^IFN-γ^−^IL-17^−^IL-22^+^ T cells, in ND, Non-CR, CR AML patients, and healthy controls is shown in Figure 1C,E,G,I. Different from one recent report [35], our results showed that the proportion of Th22 cells was significantly elevated in ND AML patients (median, 1.43% (range, 1.12%–3.98%)) relative to healthy controls (median, 0.64% (range, 0.14%–1.12%), *p* < 0.0001). Additionally, the level of Th22 cells in Non-CR AML patients (median, 1.56% (range, 1.17%–3.04%)) was also significantly increased compared with CR AML patients (median, 0.7% (range, 0.19%–0.92%), *p* < 0.0001) or healthy controls (*p* < 0.0001) (Figure 2A). That may be due to the true that they did not exclude the frequencies of Th17 and Th1 in their study. These results indicate that Th22 cells may participate in the development and progress of AML. It is interesting to note that the levels of circulating Th22 cells did not differ between patients achieving CR after chemotherapy and healthy controls (*p* = 0.718), suggesting that Th22 cells are sensitive to induction chemotherapy and that chemotherapy may play a key role in decreasing the frequencies of Th22 cells in AML. That is, increased Th22 cells may be explained as a pathogenic reaction of the immune system in patients with AML, and Th22 cells may exert a tumor-promoting effect on certain stages of the disease.

**Figure 3.**
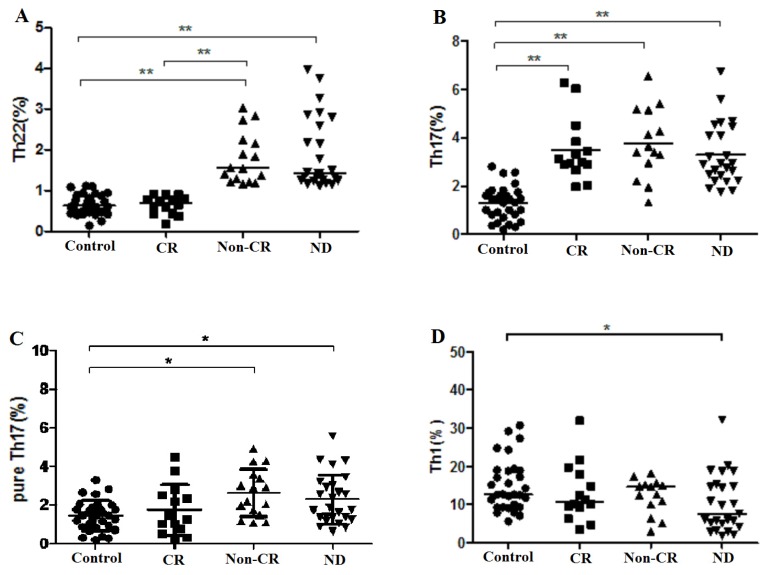
Results of cytokines in plasma from ND, Non-CR and CR AML patients and healthy controls. (**A**) Concentration of IL-22; (**B**) Concentration of IL-17. Data was shown as Median (range). * *p* < 0.05.

IL-22, a member of IL-10 family of cytokines, is produced by special immune cell populations, including CD4^+^ T cells (Th1, Th17, and Th22 cells), NK cells, NKT cells, and lymphoid tissue inducer cells [36]. IL-22 is considered as either a protective or a pathogenetic player in inflammatory disorders and autoimmune diseases [37,38]. Thus, we further investigated concentration of plasma IL-22 by Enzyme-Linked Immunosorbent Assay (ELISA). We observed a significant increase of plasma IL-22 levels in ND AML patients (median, 36.62 pg/mL (range, 14.63–3037.10 pg/mL)) compared to healthy controls (median, 26.82 pg/mL (range, 7.58–69.45 pg/mL), *p* = 0.0016) (Figure 3A), which is in accordance with the increasing percentage of Th22 cells. However, no significant increase of plasma IL-22 was found in Non-CR AML patients (median, 35.13 pg/mL (range, 7.58–49.73 pg/mL), *p* = 0.20) or CR AML patients (median, 30.90 pg/mL (range, 18.43–56.30 pg/mL), *p* = 0.17) compared with healthy controls (Figure 3A). Similarly, the level of plasma IL-22 was not significantly different between CR AML patients and Non-CR AML patients. Strangely, no correlation was found between Th22 cells and plasma level of IL-22 (*r* = 0.22, *p* = 0.33) in ND AML patients.

### 2.2. Elevated Th17 and Decreased Th1 Cells in AML Patients

In recent years, a few reports indicate that the number of Th17 cells was increased in patients with solid tumors such as advanced ovarian carcinoma and gastric cancer [39,40]. Wu *et al.* have described increased frequency of circulating Th17 cells in untreated patients with AML and normalized after complete remission [3]. In contrast, Elisabeth *et al.* observed normal Th17 levels in untreated patients with AML compared with healthy controls [1]. Here, we showed that the frequency of Th17 cells was significantly higher in ND AML patients (3.3% ± 1.28%, *p* < 0.0001), Non-CR AML patients (3.78% ± 1.46%, *p* < 0.0001), and CR AML patients (3.50% ± 1.30%, *p* < 0.0001), compared to healthy controls (1.31% ± 0.69%) (Figure 2B). However, no difference of Th17 cells was found between CR AML patients and Non-CR AML patients.

To exclude Th1 and Th22 cells, pure Th17, defined as CD4^+^IFN-γ^−^IL-22^−^IL17^+^ T cells, were also analyzed. The expression of a typical dot plot of pure Th17 cells in each group is shown in Figure 1C,E,G,I. The percentage of pure Th17 cells was significantly elevated in both ND AML patients (2.22% ± 1.28%, *p* = 0.0039) and Non-CR AML patients (2.62% ± 1.63%, *p* = 0.0002) compared to healthy controls (1.38% ± 0.70%) (Figure 2C). No difference was found between CR AML patients and healthy controls or Non-CR AML patients (*p* = 0.750, *p* = 0.793). Therefore, we deduce that Th17 and pure Th17 cells may promote the development of AML and exert a pathogenetic role in AML. In addition, Th17 and pure Th17 cells might not be affected by chemotherapy.

Strangely, we found a significant decrease of plasma IL-17 in ND AML patients (median, 2.34 pg/mL (range, 1.38–31.57 pg/mL), *p* = 0.024), Non-CR AML patients (median, 2.63 pg/mL (range, 1.04–3.34 pg/mL), *p* = 0.030) or CR AML patients (median, 2.13 pg/mL (range, 1.30–21.74 pg/mL), *p* = 0.0002) relative to healthy controls (median, 3.05 pg/mL (range, 1.46–3.40 pg/mL)) (Figure 3B). No difference was found between CR and Non-CR AML patients (*p* = 0.1349). The opposite situation of Th17 or pure Th17 cells with IL-17 demonstrated that the promoting effect of Th17 and pure Th17 cells in AML might not be due to the direct secretion of IL-17.

**Figure 4.**
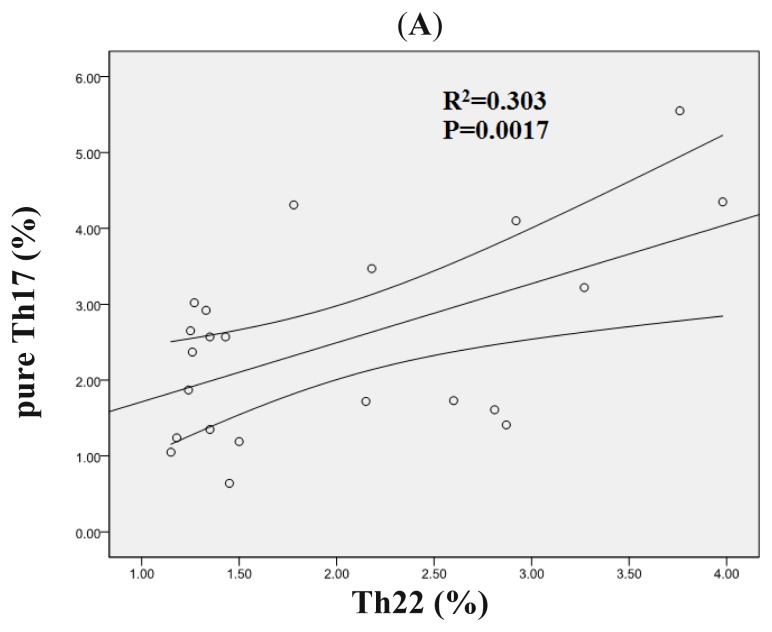

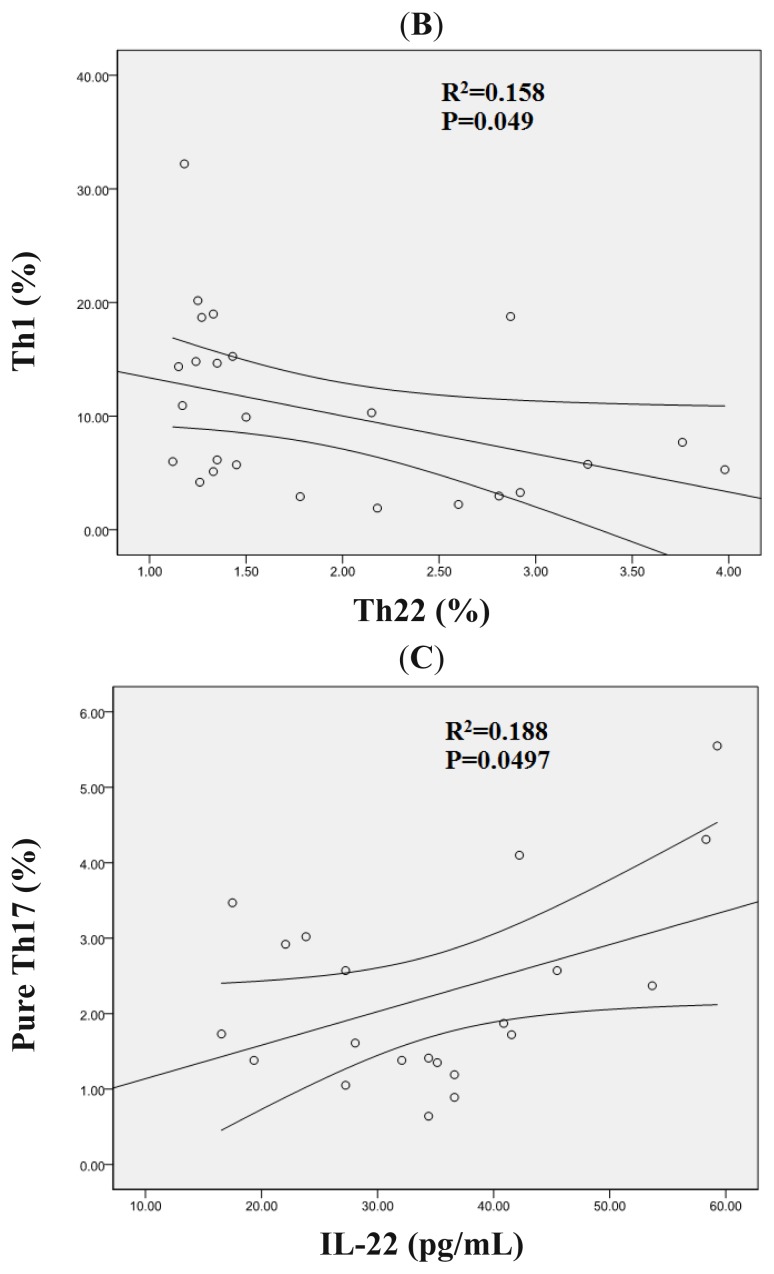
Correlations between Th subsets and cytokines in AML patients. (**A**) A positive correlation was found between Th22 cells and pure Th17 cells in ND AML patients; (**B**) A negative correlation between Th22 cells and Th1 cells in ND AML patients; (**C**) Plasma IL-22 showed a positive correlation with pure Th17 cells in ND AML patients.

Additionally, we observed that the elevated plasma concentration of IL-22 was positively correlated to the frequency of pure Th17 cells in ND AML patients (*r* = 0.43, *p* = 0.0497) (Figure 4C). However, no correlation was found between IL-22 and Th17 cells, as well as IL-17 in ND AML patients.

As for Th1 cells, there was a statistical decrease in peripheral blood from ND AML patients (10.33% ± 7.48%) compared with healthy controls (15.13% ± 6.75%, *p* = 0.016) (Figure 2D), which is in line with the significantly decreased Th1 during therapy-induced cytopenia, reported by Elisabeth *et al.* [1]. No significant difference was found between Non-CR AML patients and healthy controls, between CR patients and healthy controls, as well as between CR and Non-CR AML patients (*p* = 0.459, *p* = 0.177, *p* = 0.477).

### 2.3. Correlation Analysis between Th22, Th17, Pure Th17, and Th1 Cells in AML Patients

In ND AML patients, a statistically positive correlation was found between Th22 and pure Th17 cells (*r* = 0.60, *p* = 0.0017) (Figure 4A), which indicated that there may be a synergy between Th22 and pure Th17 cells in the pathogenesis of AML. There was a negative correlation between Th22 and Th1 cells (*r* = −0.40, *p* = 0.049) (Figure 4B). Nevertheless, No significant correlation was found between Th22 and Th17 cells in ND AML patients. No correlations between Th22, pure Th17, Th17, and Th1 cells existed in Non-CR and CR AML patients.

### 2.4. Increased Expression Profile of *RORC* in Patients with AML

**Figure 5.**
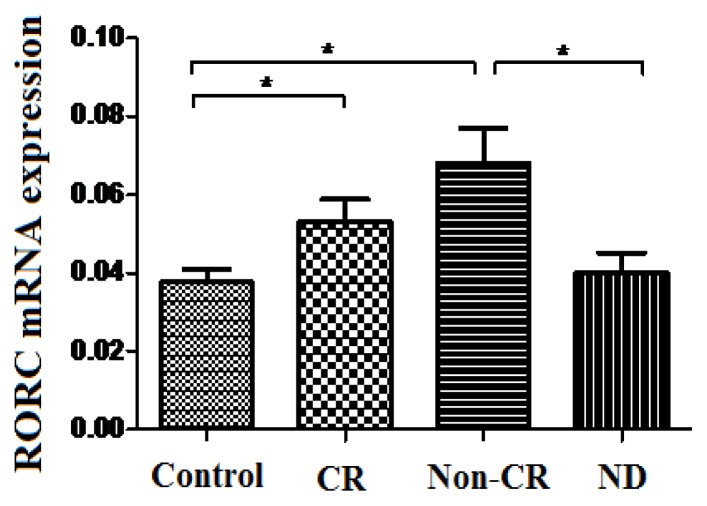
The mRNA expression of *RORC* expression in AML patients and healthy controls. The data represent the means ± SD and * *p* < 0.05.

*RORC* is important in regulating the expression of a wide variety of cytokines. Research into *RORC* expressing cells took flight with the appreciation of a role for *RORC* in directing effector T cells toward IL-17 and IL-22 producing T helper-17 cells [7]. In the current study, the expression of *RORC* was significantly increased in Non-CR AML patients (0.068 ± 0.039) compared to ND AML patients (0.04 ± 0.024, *p* = 0.007) or healthy controls (0.038 ± 0.014, *p* = 0.004) (Figure 5). The expression of *RORC* was also elevated significantly in CR AML patients (0.053 ± 0.019) compared with healthy controls (*p* = 0.019) (Figure 5). By contrast, no significant difference of *RORC* mRNA level was observed between ND AML patients and healthy controls, or between Non-CR and CR AML patients (*p* = 0.686, *p* = 0.165).

**Figure 6.**
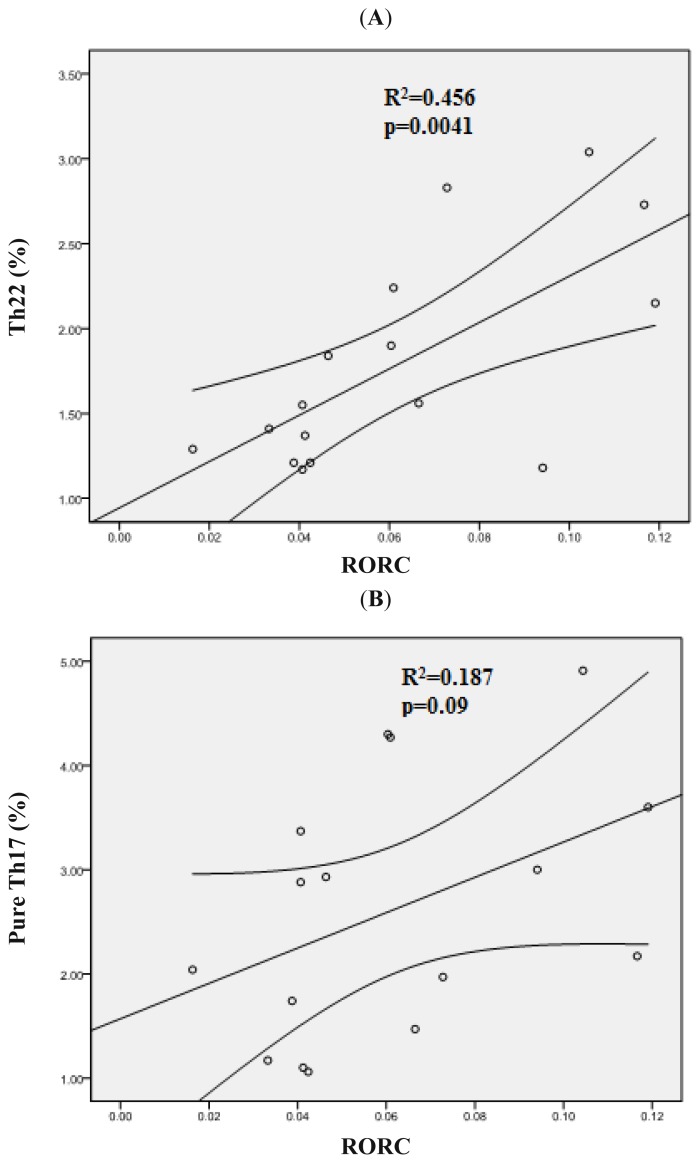
Correlations between *RORC* and Th subsets in AML patients. (**A**) *RORC* showed the positive correlation with Th22 cells in Non-CR AML patients; (**B**) *RORC* showed the negative correlation with Th1 cells in Non-CR AML patients.

Moreover, our data demonstrated that elevated *RORC* expression positively correlated with Th22 cells (*r* = 0.67, *p* = 0.0041) (Figure 6A), which seems to be reasonable to explain the increase of Th22 cells in Non-CR patients. In addition, an approximately positive correlation between *RORC* and pure Th17 cells also exists in Non-CR patients (*r* = 0.43, *p* = 0.09) (Figure 6B). These findings suggest that *RORC* appears have a crucial role in regulating the differentiation of both Th22 and pure Th17 cells in Non-CR AML patients.

### 2.5. Clinical Relevance of Th22, Th17, Pure Th17 and Th1 Cells in Patients with AML

**Figure 7.**
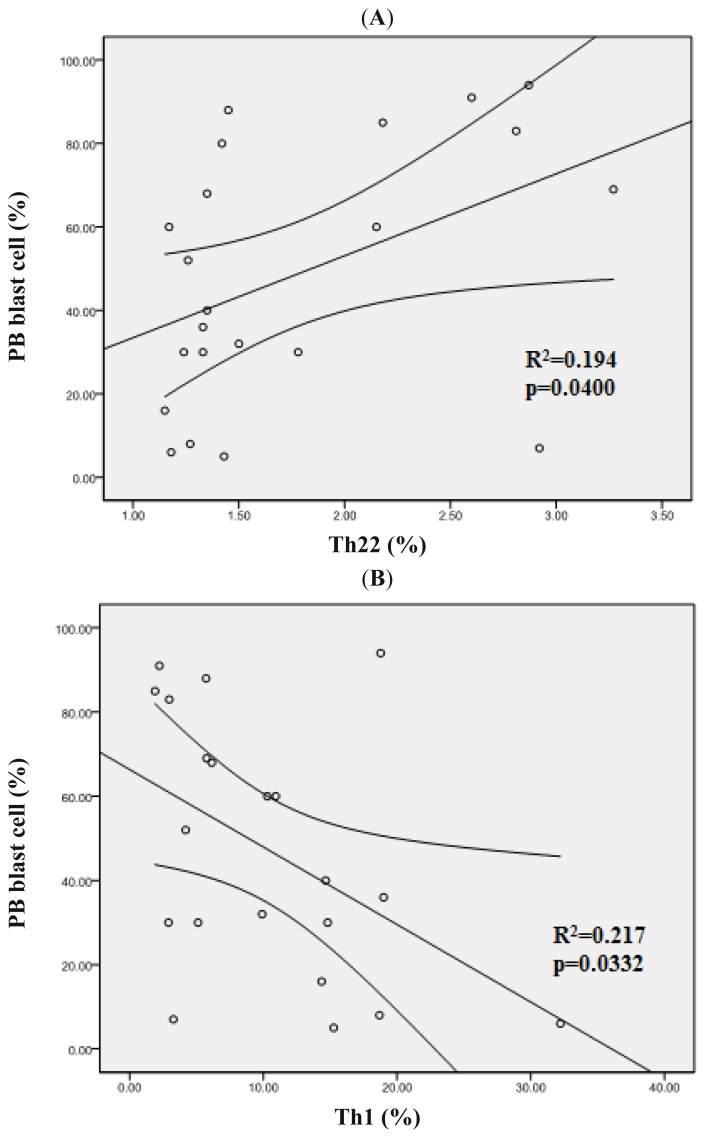
Clinical relevance of Th22 and Th1 cells in AML patients. (**A**) A positive correlation was observed between Th22 cells and PB blast cell in ND AML patients; (**B**) A negative correlation was observed between Th1 cells and PB blast cell in ND AML patients.

We analyzed the correlation between blast cell and Th subsets. In ND AML patients, the positive correlation was found between Th22 cells and PB blast cell (*r* = 0.4408, *p* = 0.04) (Figure 7A). However, Th1 cells showed a negative correlation with PB blast cell (*r* = −0.466, *p* = 0.0332) (Figure 7B). No association was observed between bone marrow (BM) blast cell and Th subsets (*p* > 0.05).

### 2.6. Increased Th22, Th17, Pure Th17 in Acute Myelomonocytic Leukemia

**Figure 8.**
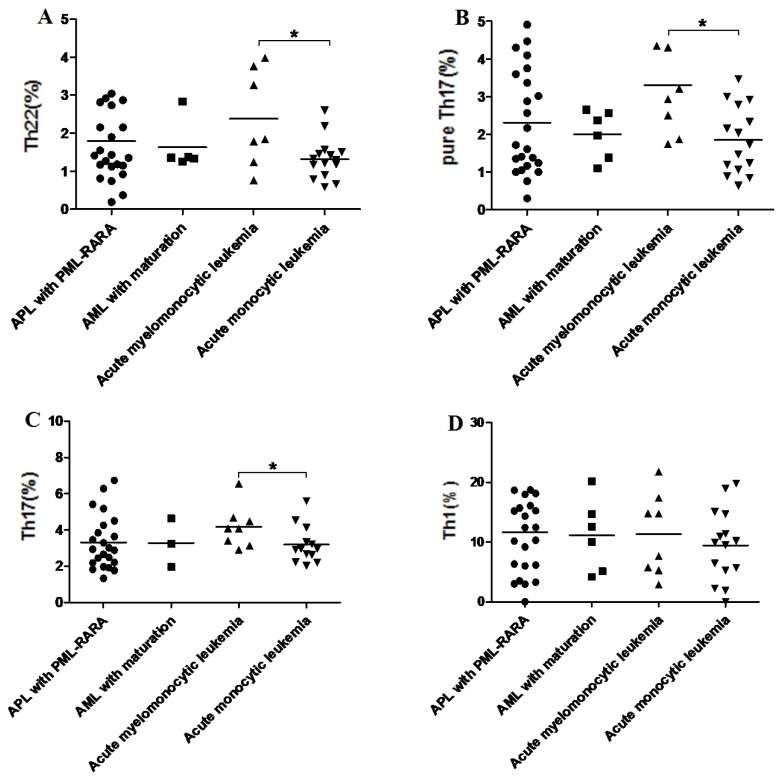
Comparison of Th subsets in different AML patients. Th22 (**A**); pure Th17 (**B**); and Th17 cells (**C**) were significantly increased in acute myelomonocytic leukemia patients compared with acute monocytic leukemia patients; (**D**) No significant difference of Th1 cells was found in different AML patients. Data was shown as Median (range). * *p* < 0.05.

We compared Th subsets from APL with PML-RARA, AML with maturation, acute myelomonocytic leukemia, and acute monocytic leukemia. As shown in Figure 8A, the frequencies of Th22 were significantly increased in acute myelomonocytic leukemia patients (median, 2.55% (range, 0.76%–3.98%)) compared with acute monocytic leukemia patients (median, 1.29% (range, 0.58%–2.18%), *p* = 0.0365). Moreover, Th17 and pure Th17 cells were also higher in acute myelomonocytic leukemia patients (4.08% ± 1.15%; 3.07% ± 1.33%) than acute monocytic leukemia patients (3.18% ± 1.02%, *p* = 0.0463; 1.85% ± 0.89%, *p* = 0.0118) (Figure 8B,C). However, no significant difference of Th1 cells was found in different AML patients (*p* > 0.05) (Figure 8D).

Tumor-associated immune suppression can lead to defective T-cell-mediated immunity. In the current study, abnormal Th22, Th17, and pure Th17 cells were identified in different stages of AML patients. Recently, the PD-1 gene has been shown to be expressed at high levels by activated CD4^+^ T cells of leukemia. Moreover, PD-1 can interact with the PD-L1 expressed highly in AML cells contributing to functional T-cell impairment. Increasing data indicate that the PD-1-PD-L1 signaling pathway is related to the immune suppression and disease progression [33]. Therefore, we deduce that increased Th22, Th17, and pure Th17 cells may participate in the pathogenesis and progress of AML by PD-1-PD-L1 pathway. Of course, further study is required to explore the mechanism. Furthermore, T-cell immunodeficiency in AML may be also associated with different causes of immune suppression, such as alterative expression of T cell receptor (TCR) signaling pathway members [34].

Additionally, B cell may also play a pivotal role in the pathogenesis of leukemia. Recently, CD19, though a biomarker for B cells, has been observed in cases of myeloid malignancies, including in 2% of AML cases [41]. The B-cell leukemia/lymphoma 11B (*BCL11B*), a tumor suppressor gene, plays a key role in both T-cell development and subsequent maintenance of T-cell identity [42]. Thus, to improve the dysfunction of immunity of AML patients, further work was necessary to explore the role of T cells and B cells in AML.

## 3. Experimental Section

### 3.1. Patients and Controls

Twenty-six ND AML patients (15 females and 11 males; age range, 19–77 years; median, 39 years), 14 morphologic complete remission (CR) AML patients (9 females and 5 males; age range, 13–57 years; median, 37 years) and 16 morphologic non-complete remission (Non-CR) AML patients (5 females and 11 males; age range, 15–74 years; median, 43 years) were included in this study. AML was diagnosed according to the World Health Organization (WHO) classification system [43]. Morphologic complete remission (CR) was defined based on International Working Group criteria [44]. ND patients with non-APL (acute promyelocytic leukemia) AML subgroups underwent standard induction chemotherapy with one of the anthracyclines (daunorubicin, homoharringtonine, or idarubicin) for 3 days and cytarabine for 7 days. ND patients with APL received all-trans retinoic acid with or without concurrent induction chemotherapy. Thirty healthy volunteers (16 females and 14 males; age range, 25–58 years; median, 33 years) were studied simultaneously as controls. The demographic and key clinical information of AML patients are summarized in Table 1. The treatment regimens of AML patients were shown in Table 2. This study was approved by the Institutional Review Boards of Qilu Hospital of Shandong University. The blood samples from all the enrolled participants were obtained immediately after they admitted to our hospital and signed the informed consent.

### 3.2. Flow Cytometric Analysis

Intracellular cytokines were studied by flow cytometry to identify the cytokine-producing cells. Briefly, heparinized peripheral whole blood (400 μL) with an equal volume of Roswell Park Memorial Institute 1640 medium was incubated for 4 h at 37 °C, 5% CO_2_ in the presence of 25 ng/mL of phorbol myristate acetate (PMA), 1 μg/mL of ionomycin, and 1.7 μg/mL Golgiplug (monensin; all from Alexis Biochemicals, San Diego, CA, USA). PMA and ionomycin are pharmacologic T cell-activating agents that mimic signals generated by the T cell receptor (TCR) complex and have the advantage of stimulating T cells of any antigen specificity. Monensin is used to block intracellular transport mechanisms, thereby, leading to an accumulation of cytokines in the cells.

After incubation, the cells were stained with PE-Cy5-conjugated anti-CD4 monoclonal antibody at room temperature in the dark for 20 min. After surface staining, the cells were next stained with FITC-conjugated anti-IFN-γ, PE conjugated anti-IL-17, and APC conjugated anti-IL-22 monoclonal antibodies after fixation and permeabilization. All the antibodies were from eBioscience (San Diego, CA, USA). Isotype controls were given to enable correct compensation and confirm antibody specificity. Stained cells were analyzed by flow cytometric analysis using a FACS Calibur cytometer equipped with CellQuest software (BD Bioscience PharMingen, San Jose, CA, USA). For analysis, we first gated CD4^+^ lymphocytes, then analyzed the proportion of Th22 (CD4^+^IFN-γ^−^IL-17^−^IL-22^+^), Th17 (CD4^+^IL-17^+^) or Th1 (CD4^+^IFN-γ^+^) cells in CD4^+^ lymphocytes.

### 3.3. IL-22 and IL-17 Enzyme-Linked Immunosorbent Assay (ELISA)

Peripheral blood was collected into heparin-anticoagulant vacutainer tubes. All plasma specimens were obtained from all subjects by centrifugation and stored at −80 °C for determination of cytokines. Plasma IL-22 and IL-17 levels in each group were determined with a quantitative sandwich enzyme immunoassay technique in accordance with the manufacturer’s recommendations (eBioscience, San Diego, CA, USA). The lower detection limits were as follows: IL-22, 5 pg/mL; IL-17, 0.5 pg/mL.

### 3.4. Quantitative Real-Time PCR Analysis

Total RNA was extracted with Trizol (Invitrogen, Carlsbad, CA, USA) according to the manufacturer’s instructions. Approximately, 1 μg of total RNA from each sample was used to synthesize cDNA with PrimeScript RT reagent Kit (Takara Bio Inc., Dalian, China). Reverse transcription reaction was done at 37 °C for 15 min, followed by 85 °C for 5 s. Real-time quantitative PCR was conducted using an ABI Prism 7500 Real-time PCR system (Applied Biosystems, Foster City, CA, USA) in accordance to the manufacturer’s instructions. The real-time PCR contained, in a final volume of 20 μL, 10 μL of 2× SYBR Green Real-time PCR Master Mix, 2 μL of cDNA, and 1.6 μL of the forward and reverse primers. The primers were shown as bellows: *RORC* Forward 5′-TTTTC CGAGGATGAGATTGC-3′, Reverse 5′-CTTTCCACATGCTGGCTACA-3′; β-actin Forward 5′-CCTTC CTGGGCATGGAGTCCTG-3′, Reverse 5′-GGAGCAATGATCTTGATCTTC-3′. All experiments were conducted in triplicate. The PCR products were analyzed by melt curve analysis and agarose gel electrophoresis to determine product size and to confirm that no by-products were formed. The results were expressed relative to the number of β-actin transcripts used as an internal control.

### 3.5. Statistical Analysis

The results were expressed as median (range) or mean ± SD. Statistical significance was determined by analysis of variance (ANOVA), and Newman-Kuels multiple comparison test was used for data with a normal distribution. Kruskal-Wallis and Nemenyi tests were used for non-normal data. The Pearson or Spearman correlation test was used for correlation analysis depending on data distribution. All tests were performed by SPSS 17.0 system (IBM, Armonk, NY, USA). *p* value less than 0.05 was considered statistically significant.

## 4. Conclusions

Taken together, our findings suggest that AML individuals present increased frequencies of Th22, Th17 and pure Th17 cells and decreased Th1 cells in different stage of AML patients. The elevated Th22 cells and IL-22 showed a positive correlation with pure Th17 cells and a negative correlation with Th1 cells in ND AML patients. Therefore, it is not difficult to deduce that Th22 and pure Th17 cells conjointly contribute to the pathogenesis of AML, and Th22 might be a novel biomarker to assess patients at risk.

## Figures and Tables

**Table 1.** Clinical features for acute myeloid leukemia (AML) patients.

	ND AML patients (*n* = 26)	Non-CR AML patients (*n* = 16)	CR AML patients (*n* = 14)
Age (year)	39 (19–77)	43 (15–74)	37 (13–57)
Gender (male/female)	11/15	11/5	5/9
PB blast cell (%)	48.64 ± 30.61	34 ± 34.53	0
BM blast cell (%)	76.25 ± 17.78	47.33 ± 28.52	0.5 (0–2)
WHO classification			
APL with t (15; 17) (q22; q12); PML-RARA	11	5	7
AML with t (8; 21) (q22; q22)	2	0	0
AML, not otherwise specified			
AML without maturation	0	1	0
AML with maturation	4	2	0
Acute myelomonocytic leukemia	5	2	1
Acute monocytic leukemia	7	4	4
Acute erythroid leukemia	0	1	0

ND, newly-diagnosed; CR, complete remission; WHO, World Health Organization; PB, peripheral blood; BM, bone marrow. All data are expressed as median (range) or mean ± SD in this table.

**Table 2.** Treatment regimens of AML patients.

Treatment regimens	Number of samples
ATRA	4
ATRA and DNR	2
HA	1
IA	1
Multiple cycles of chemotherapy	22

ATRA, all-trans retinoic acid; DNR, daunorubicin; HA, cytarbine and homoharringtonine; IA, cytarbine and idarubine.
